# Hidden Malignancy: Predominantly *In* *Situ* Porocarcinoma Arising in a Poroma

**DOI:** 10.1097/DAD.0000000000003400

**Published:** 2026-08-20

**Authors:** Fabio Del Carro, Giovanni Evalli, Giovanni Fellegara

**Affiliations:** * School of Medicine and Surgery, University of Milan-Bicocca, Monza, Italy;; † Pathology Unit, Centro Diagnostico Italiano (CDI), Milan, Italy; and; ‡ Centro Salute Colleoni, Castano Primo, Italy.

**Keywords:** porocarcinoma, poroma, cutaneous adnexal tumors, skin cancer, mismatch repair proteins

## Abstract

Porocarcinoma is a rare malignant adnexal tumor which can originate either *de novo* or—much more rarely—from a preexisting eccrine poroma. Given its aggressive clinical behavior, a timely diagnosis is essential for optimal patient's management. We report the case of a 79-year-old woman presenting with a well-circumscribed exophytic mass located on her right calcaneal region, which had been present for 2 years but had recently shown progressive enlargement. An excisional diagnostic biopsy was performed, and histopathological examination showed a poroid lesion with no malignant features. A diagnosis of eccrine poroma was then considered. However, deeper sections revealed a focal intraepidermal proliferation of markedly atypical cells and a small intradermal nest suggestive of early dermal invasion. The atypical population was characterized by overexpression of both p53 and p16, an increased ki-67 proliferation index, and preserved mismatch repair proteins. The diagnosis was then changed into a predominantly *in situ*, focally invasive porocarcinoma arising in an eccrine poroma. The patient underwent electrocautery of the wound margins and an echographic examination of pelvic nodes—which were negative for metastases—and was free of recurrence at a 6-month follow-up. This case provides evidence that benign-looking eccrine poromas may harbor microscopic foci of malignant transformation, thus emphasizing the need for a careful and thorough histopathological evaluation of these lesions.

## INTRODUCTION

Porocarcinoma is a malignant adnexal tumor originating from the intraepidermal portion of eccrine sweat glands (*acrosyringium*). It typically arises on the extremities of older patients, either *de novo* or, much more rarely, from a preexisting eccrine poroma, of which it is regarded as the malignant counterpart.^1,2^ Although a rare tumor, it has quite an aggressive behavior, with reported nodal and distant metastasis rates up to about 20% and 15%, respectively, and an associated 5-year mortality rate higher than 30%.^3^ Thus, a prompt and accurate diagnosis is of paramount importance for optimal patient's management. Since dermoscopy can be misleading, the diagnostic gold standard is represented by histopathological evaluation with standard Hematoxylin & Eosin—which shows ductal differentiation, nuclear atypia, increased mitotic rate and, possibly, foci of necrosis—and immunohistochemistry—which typically demonstrates abnormal expression of tumor-suppressor genes such as p53.^3,4^ The first-line treatment is complete surgical excision, whenever feasible, typically with Mohs micrographic surgery. For advanced cases, systemic chemotherapy and radiotherapy can be considered.^5^ In this report, we present the case of an eccrine poroma harboring scattered foci of malignancy, warning against this rare, sneaky histological pattern of porocarcinoma which may easily go undetected, and thus requires thorough, careful evaluation of the entire lesion.

## CASE REPORT

**FIGURE 1.**
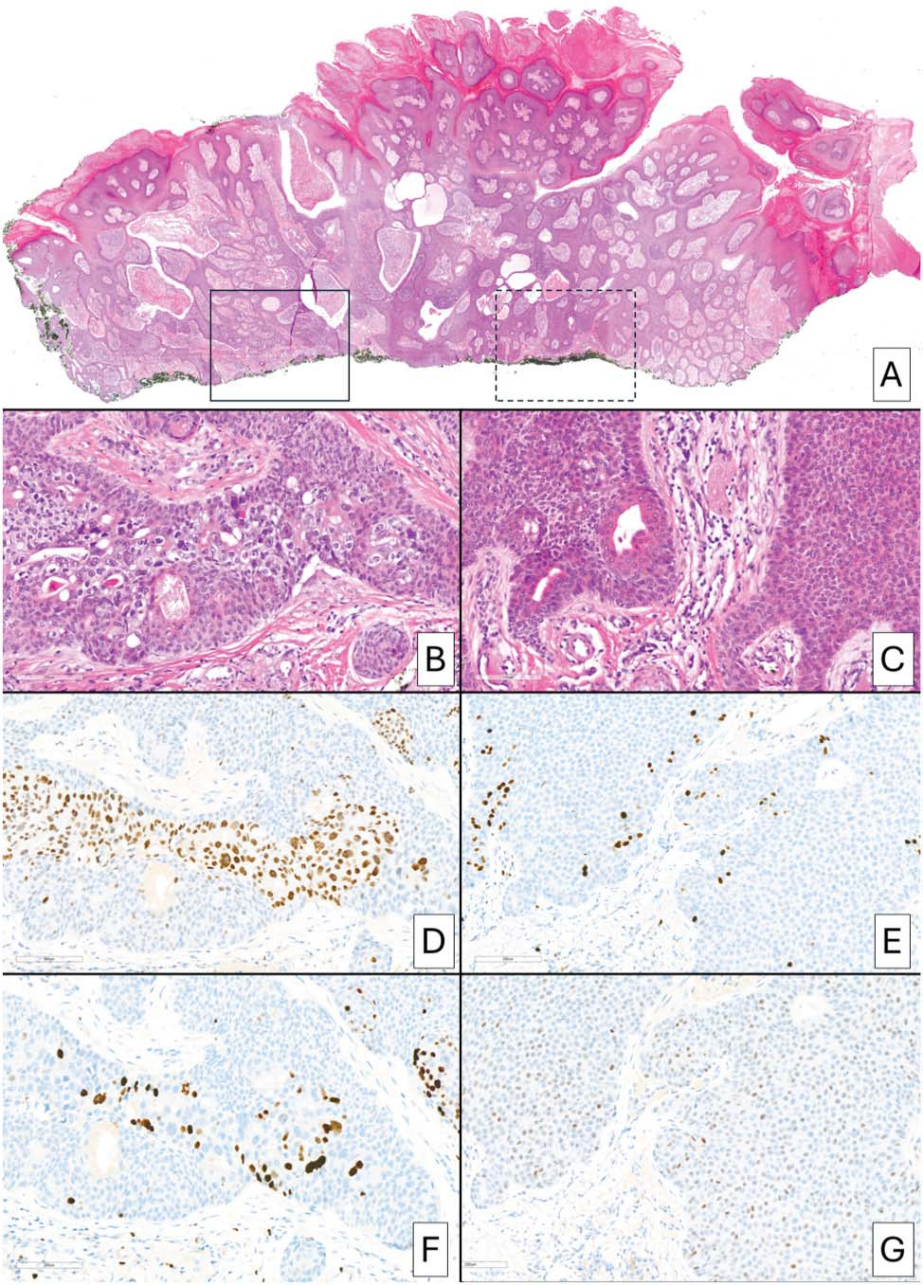
Low-power view shows a hyperkeratotic cutaneous lesion with combined exophytic and endophytic growth, composed of anastomosing cords of poroid-appearing cells with focal ductal differentiation (solid rectangle in A [Hematoxylin and Eosin; ×20], higher magnification in C [Hematoxylin and Eosin; ×100]). In the deeper portion of the lesion, aggregates of atypical cells are identified (dashed rectangle in A, higher magnification in B [Hematoxylin and Eosin; ×100]). These cells show an increased proliferation index by ki-67 (D; ×100) and p53 overexpression (F; ×100) as compared with the benign cellular component (E, G; both ×100).

**FIGURE 2.**
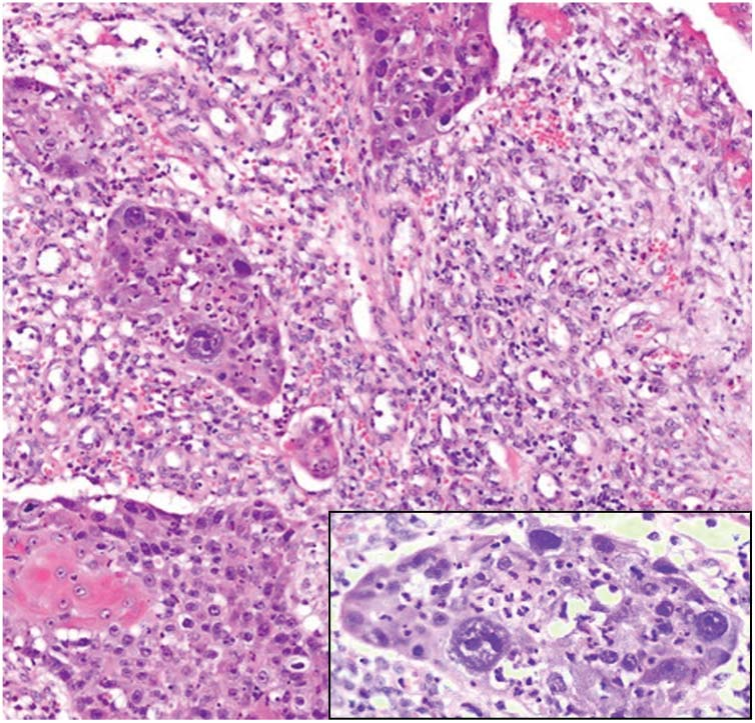
An aggregate of highly atypical cells associated with brisk inflammatory infiltrate and prominent granulocytic exocytosis is identified within the derma, raising suspicion for early stromal invasion (Hematoxylin and Eosin; ×100, inset ×400).

A 79-year-old White woman with no significant medical history presented to our surgical outpatient clinic with a painless, well-circumscribed, 30-mm exophytic mass located on her right calcaneal region. The lesion had been present for approximately 2 years and had recently shown progressive enlargement, leading to discomfort. On clinical examination, the surface was intact, with no signs of ulceration or spontaneous bleeding. A diagnostic surgical excision was then performed. Histopathological examination revealed a hyperkeratotic cutaneous lesion with both an exophytic and an endophytic growth pattern (Fig. 1A), characterized by a predominantly intraepidermal proliferation of uniform, cytologically bland cells with round nuclei and scant cytoplasm, arranged in cords and anastomosing bridges extending from and connected to the epidermis. Focal areas suggestive of ductal differentiation were also noticed, whereas there was no evidence of necrosis (Fig. 1C). Although a diagnosis of eccrine poroma was initially considered, examination of deeper sections revealed a focal intraepidermal proliferation of markedly atypical cells, characterized by an increased nuclear-to-cytoplasmic ratio, pronounced nuclear pleomorphism, and atypical mitotic figures (Fig. 1B). This atypical component was confined to the deep portion of the lesion and was contiguous with a small intradermal nest of similarly atypical cells, suggestive of early dermal invasion (Fig. 2). In addition, other small intraepidermal foci of atypical cells were identified scattered throughout the lesion. On immunohistochemistry, the atypical cells showed overexpression of both p53 and p16, as compared with the typical poroid cells, along with an increased ki-67 proliferation index (Figs. 1D–G). The expression of mismatch repair proteins was preserved in both the components. Overall, the morphological and immunohistochemical findings were consistent with a diagnosis of predominantly *in situ*, focally invasive porocarcinoma arising in an eccrine poroma. Since surgical radicalization could not be reached because of the site and size of the lesion, electrocautery of the wound margins was performed, as well as an echographic examination of the patient's inguinal lymph nodes—which were negative for metastases. The patient was free of local recurrence at a 6-month follow-up, and the surgical wound healing was unremarkable.

## DISCUSSION

Porocarcinoma is an uncommon malignant adnexal tumor of eccrine origin arising either *de novo* or, much more rarely, from a preexisting eccrine poroma.^1^ It is an aggressive, potentially fatal disease, whose diagnosis is not infrequently delayed because of deceiving clinical presentation.^3^ As a consequence, *in situ* porocarcinomas are only rarely encountered in the real-life routine setting, in favor of more advanced scenarios with overtly invasive lesions,^6^ and their association with an eccrine poroma is anecdotical.^7^ In this study, we reported a peculiar, paradigmatic case of a single lesion embracing the full spectrum of eccrine neoplasms—a predominantly *in situ*, focally invasive porocarcinoma arising in an eccrine poroma—providing evidence that eccrine poromas with an otherwise benign histopathological appearance may harbor microscopic foci of malignant transformation, and thus emphasizing the need for a careful and thorough histopathological evaluation. More specifically, since the malignant component may be confined to the deeper portions of the lesion, superficial sampling could lead to underestimation of the diagnosis. In this scenario, immunohistochemical studies can serve as an important diagnostic tool.^3,4^ Additionally, our observations indicate that mismatch repair protein deficiency is unlikely to contribute to the earliest stages of poroma-to-porocarcinoma progression.
